# Ovarian Sex Cord Stromal Tumor in a Free-Ranging Brown Bear (*Ursus arctos*)

**DOI:** 10.3390/ani14131936

**Published:** 2024-06-30

**Authors:** Natalia García-Álvarez, Álvaro Oleaga, María José García-Iglesias, Claudia Pérez-Martínez, Daniel Fernández, Luis Miguel Álvarez, Ramón Balsera, Ana Balseiro

**Affiliations:** 1 Departamento de Sanidad Animal, Facultad de Veterinaria, Universidad de León, 24071 León, Spain; ngara@unileon.es (N.G.-Á.); mjgari@unileon.es (M.J.G.-I.); cperm@unileon.es (C.P.-M.); 2 Sociedad de Servicios del Principado de Asturias S.A. (SERPA), La Laboral, 33203 Gijón, Spain; alvaroleaga@yahoo.es (Á.O.); danielfernandez.vet@gmail.com (D.F.); 3 Dirección General de Planificación Agraria del Principado de Asturias, 33007 Oviedo, Spain; luismiguel.alvarezmorales@asturias.org (L.M.Á.); ramon.balserariesgo@asturias.org (R.B.); 4 Departamento de Sanidad Animal, Instituto de Ganadería de Montaña (CSIC-ULE), Finca Marzanas, Grulleros, 24346 León, Spain

**Keywords:** brown bear (*Ursus arctos*), sex cord stromal tumor, steroid cell tumor, ovary, pathology, immunohistochemistry

## Abstract

**Simple Summary:**

Here, we describe an unusual metastatic ovarian sex cord stromal tumor in a 14-year-old free-ranging female Eurasian brown bear (*Ursus arctos*) from Northwestern Spain. Based on histopathological and immunohistochemical studies, the tumor was diagnosed as a steroid cell tumor, not otherwise specified.

**Abstract:**

Reports on neoplasms in bears are scarce, especially concerning ovarian tumors. A large primary ovarian neoplasm with multiple metastasis was found during the necropsy of a 14-year-old free-ranging Eurasian brown bear (*Ursus arctos*) from Northwestern Spain. Histopathology and immunohistochemistry allowed for the diagnosis of a sex cord stromal tumor. This is a complex group of neoplasms which differ in the predominant cell morphology and immunohistochemical features. The microscopic examination revealed two types of cells, one with eosinophilic cytoplasm, intermingled with larger vacuolated cells rich in lipids. The evaluation of the immunoreactivity to different markers, frequently used in the characterization of gonadal tumors (INHA, inhibin-alpha; PLAP, placental alkaline phosphatase; Ki-67; α-SMA, actin alpha-smooth muscle) and inflammation patterns (IBA1, ionized calcium-binding adapter molecule for macrophages; CD3 for T lymphocytes; CD20 for B lymphocytes), displayed significant INHA positive immunostaining of neoplastic cells, as well as inflammatory cell infiltration, mainly composed of macrophages and B lymphocytes. These findings were consistent with a malignant ovarian steroid cell tumor, not otherwise specified. The present study characterizes an unusual type of neoplasm, and also represents the first report of an ovarian sex cord stromal tumor in *Ursidae*.

## 1. Introduction

There are few reports of neoplasms in bears, especially in free-ranging individuals [1,2,3], and only one concerning ovarian tumor in a captive bear, in which a carcinoma was diagnosed [4]. Granulosa and a theca cell tumor were also found in a giant panda (*Ailuropoda melanoleuca*) [5].

Ovarian neoplasms involve an extremely wide variety of tumors that include epithelial, sex cord stromal, and germ cell tumors [6,7]. Although sex cord stromal tumors (SCSTs) represent less than 10% of all human primary ovarian tumors [8], they are more frequently described in other animal species [7,9], accounting for two-thirds of ovarian tumors reported in cattle [10]. SCSTs comprise a heterogeneous group of neoplasms, with the following classification according to the World Health Organization [8,11]: (i) pure stromal tumors (fibroma, fibrosarcoma, thecomas, Leydig, steroid, or luteinized cell tumors); (ii) pure sex cord tumors (granulosa and Sertoli cell tumors); and (iii) mixed sex cord stromal tumors (Sertoli–Leydig cell tumors). Due to its complexity, a recent review has classified the SCSTs into five groups based on the predominant neoplastic cell morphology and immunohistochemical features [12]: Group 1: predominance of fibromatous/thecomatous cells and/or stromal cells of unusual morphology; Group 2: predominance of steroid or luteinized cells; Group 3: predominance of follicular cells; Group 4: predominance of Sertoli cells; and Group 5: predominance of sarcomatoid/unclassified/poorly differentiated cells.

As stated in [12], besides morphological study, the immunohistochemical characterization of neoplastic cells is a valuable tool for the neoplasm diagnosis, i.e., the combined use of primary antibodies allows for the identification of the tumor histotype [13]. Thus, epithelial ovarian tumors express cytokeratins (CK), germ cell tumors placental alkaline phosphatase (PLAP), and SCSTs inhibin-alpha (INHA), a peptide hormone produced by ovarian cells to inhibit follicle-stimulating hormone (FSH) [6,14,15]. Indeed, INHA is applied to discern sex cord stromal tumors from other primary ovarian tumors [13,14,15].

The aim of the present study was to describe an ovarian tumor with multiple metastases which was found in a free-ranging, 14-year-old female brown bear (*Ursus arctos*). Histopathological and immunohistochemical features enabled us to diagnose and characterize an unusual type of ovarian SCST, namely, steroid cell tumor (SCT), not otherwise specified (NOS), which, to the authors’ knowledge, represents the first report of an SCST in *Ursidae*.

## 2. Materials and Methods

### 2.1. The Brown Bear

**Figure 1:**
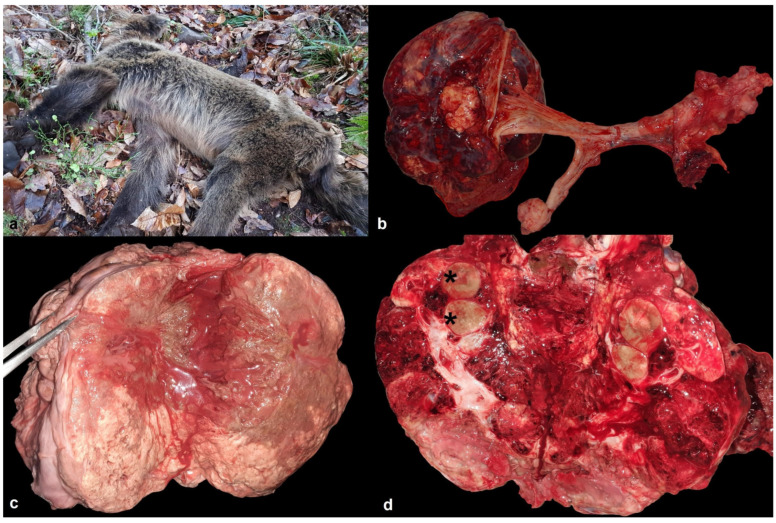
Steroid cell tumor, brown bear (*Ursus arctos*). (**a**) The 14-year-old female shows emaciation. (**b**) Neoplastic growth can be observed in the left (20 cm in diameter) and right (5 cm in diameter) ovaries. The masses are solid, with white to yellow-brown areas, and also show hemorrhages. (**c**) Necrosis and hemorrhage are observed on a section of left ovary. (**d**) Left kidney (20 cm in diameter) has lost the normal structure on section, and only two reniculi are remaining (asterisks).

A free-ranging female Eurasian brown bear was found dead in the field by official rangers in January 2024 in the Principality of Asturias (Northwestern Spain). The bear showed very poor body condition and emaciation, weighing 56 kg (Figure 1a); on average, an adult female brown bear weighs 100 kg to 140 kg, with fluctuations during the year [16]. The specimen was transported to the Wildlife Rehabilitation Center of Asturias and preserved at 4 °C before being submitted to necropsy examination.

### 2.2. Diagnostic Procedures

Necropsy was performed within less than 24 hours, and samples for histopathological evaluation were taken from the ovaries, peritoneum, diaphragm, lungs, kidneys, adrenal glands, liver, spleen, heart, gastrointestinal tract, central nervous system, skeletal muscles (femoral and *longissimus dorsi*), and lymph nodes. Samples were fixed in 10% neutral-buffered formalin and processed routinely. Serial sections (2.5 µm) were cut from different tissues for histological assessment by routine hematoxylin–eosin (HE) staining, as well as Masson’s Trichrome stain. In addition, Sudan red stain was used in frozen ovarian sections (15 µm) to detect fat droplets. A dental histological study of the first premolar was conducted for age determination [17].

Different immunohistochemical procedures were also performed to diagnose the type of tumor, according to the manufacturer’s specifications and following previously described methods [3] (main details displayed in Table 1). Briefly, tissue sections were deparaffinized and heated in sodium citrate buffer (pH 6) for antigen retrieval. To inactivate endogenous peroxidase (H_2_O_2_), the sections were exposed to 0.5% H_2_O_2_ in distilled water for 30 min. Then, the slides were incubated with 5% of goat serum (for polyclonal antibodies) or horse serum (for monoclonal antibodies) in tris-buffered saline (TBS) and bovine serum albumin (BSA) 0.1% to prevent non-specific binding. Subsequently, they were incubated overnight at 4 °C with different primary antibodies diluted in TBS and BSA 0.1% (Table 1). Afterwards, the samples were exposed to an anti-rabbit (Vector Laboratories, Newark, CA, USA, for polyclonal primary antibodies) or anti-mouse (Vector Laboratories, Newark, CA, USA, for monoclonal primary antibody) secondary antibody diluted 1:200 in TBS and BSA 0.1%, as appropriate. The slides were incubated for 40 min with the avidin–biotin–peroxidase complex reagent (ABC Standard, Vector Laboratories, Newark, CA, USA) in TBS (1×), and NovaRed (Vector Laboratories, Newark, CA, USA) was used as the chromogen. Finally, the tissue sections were stained with hematoxylin, dehydrated, and assembled with DPX (Fluka, Sigma, Saint Louis, MO, USA).

Positive and negative controls were applied in each immunohistochemical protocol. Slides without primary antibodies were used as negative controls as indicators of potential non-specific reactions, and positive tissue controls were selected based on the specific procedure (see Table 1).

## 3. Results

### 3.1. Gross Findings

Macroscopic examination showed hemoperitoneum, as well as multiple tumors in the ovaries, peritoneum, kidneys, spleen, liver, visceral side of diaphragm, and right bronchial lymph node in the thoracic cavity. Macroscopically, the anomalous neoplastic-like growth of the left ovary was irregular, measuring 20 cm in diameter and weighing 2100 g (Figure 1b). The tumor observed in the right ovary was 5 cm in diameter (Figure 1b). The masses were solid and firm, with white to yellow-brown areas. Necrosis and hemorrhage phenomena were also observed on the sections (Figure 1c). The extraovarian spread of the tumor was mainly noted in the peritoneum, which showed numerous and widespread nodules of about 0.5–1 cm in diameter, and the left kidney, which had almost lost all its normal structure, measured 20 cm and weighed 1562 g (Figure 1d). The right kidney had a few white miliary lesions in several reniculi. The spleen showed two masses of approximately 10–12 cm, both of firm consistency, hemorrhagic, and without well-defined structure. The liver parenchyma displayed multifocal white nodules of 0.2–3 cm in diameter, mainly on its diaphragmatic side. On the visceral side of the diaphragm, a 10 cm whitish growth was also observed. The right bronchial lymph node was enlarged and measured 7 cm in diameter.

### 3.2. Microscopic Features

**Figure 2:**
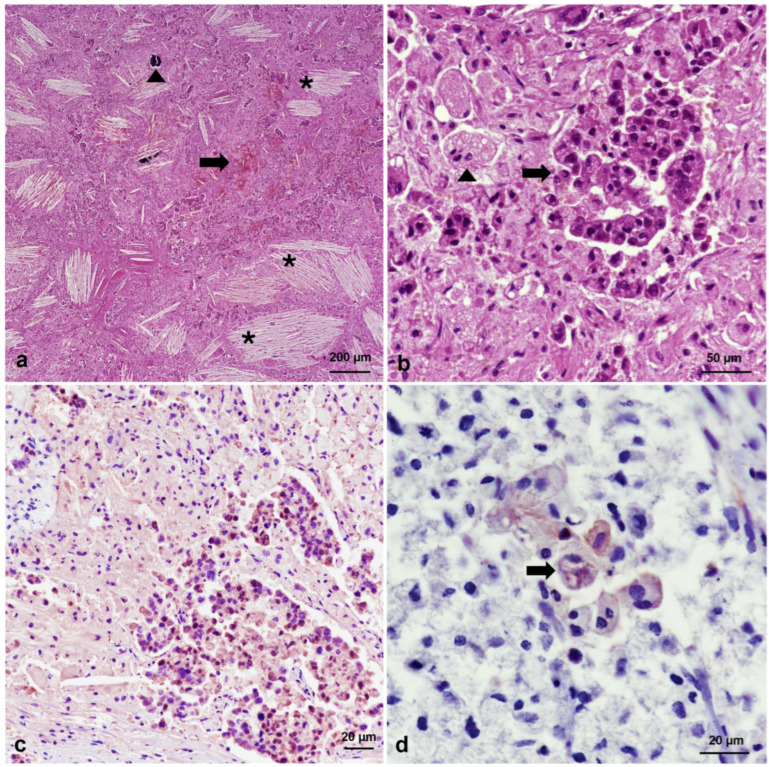
Histopathological and immunohistochemical features of a steroid cell tumor in a brown bear (*Ursus arctos*). (**a**) Left ovary. Histological image of the tumor, in which crystals of cholesterol (asterisks), necrosis, hemorrhage (arrow), and calcification (arrowhead) can be observed. Hematoxylin and eosin (HE), ×40 magnification. (**b**) Left ovary. Detail of the two types of cells observed. Aggregates of round cells with eosinophilic cytoplasm (arrow) are intermingled with larger cells containing multivacuolated or spongy cytoplasm (arrowhead). HE, ×400 magnification. (**c**) Left ovary. Neoplastic cells showing inhibin-alpha (INHA) cytoplasmic immunoreactivity. ABC complex, ×200 magnification. (**d**) Right ovary. Subset of multinucleated giant neoplastic cells with reactivity to anti-placental alkaline phosphatase (PLAP) antibody (arrow). ABC complex, ×400 magnification.

Microscopic examination revealed that tumors in all tissues showed neoplastic cells arranged in a diffuse pattern, but they also formed aggregates and small nests within a vascularized stromal tissue, sometimes exhibiting areas of calcification (Figure 2a) and inflammation. Two types of round-to-polygonal cells were observed, one with round nuclei and prominent nucleoli with abundant granular eosinophilic cytoplasms, which were intermingled with larger cells with small nuclei and multivacuolated or spongy pale cytoplasms. These latter cells were also rich in lipids, as confirmed by Sudan red stain, similar to adrenocortical cells (Figure 2a,b). Cell pleomorphism, anisocytosis, and anisokaryosis were marked, and numerous binucleated and multinucleated giant neoplastic cells were identified, as were vascular invasion and high mitotic figures, i.e., 7 mitosis per 10 high-power fields (HPFs) at ×400 magnification (2.37 mm^2^). Crystals of cholesterol, hemorrhages, and necrosis were consistently present in the tumor masses (Figure 2a). No relevant lesions were observed in other tissues or organs. The dental histological study revealed that the bear was 14 years old.

**Figure 3:**
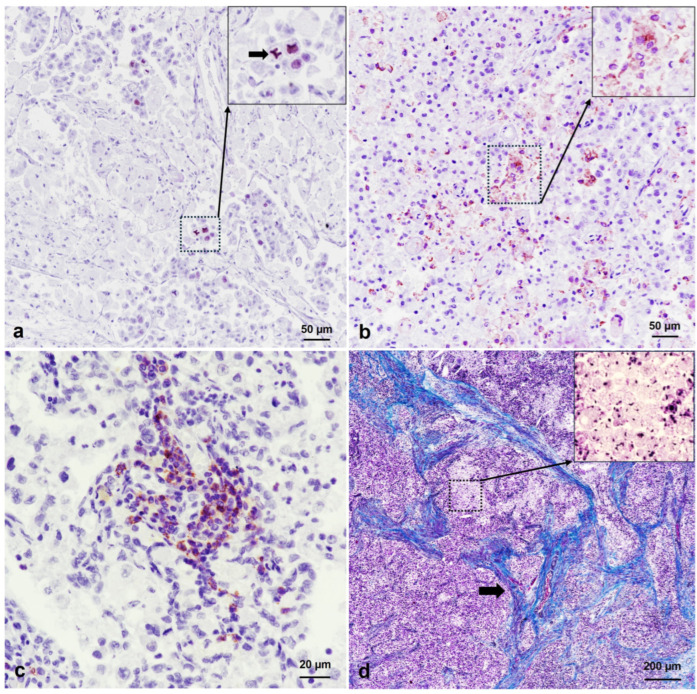
Immunohistochemical features of a steroid cell tumor in a brown bear (*Ursus arctos*). (**a**) Left ovary. Tumor proliferation is observed after Ki-67 immunostaining. ABC complex, ×200 magnification. Inset: Ki-67-positive cells are highlighted, and atypic mitosis is shown (arrow). (**b**) Right bronchial lymph node metastasis. IBA1 immunostained macrophages and multinucleated giant cells in the inflammatory infiltrate of the tumor are observed. Inset: Higher magnification of immunostained macrophages. Note that multinucleated giant neoplastic cells do not express IBA1. ABC complex, ×200 magnification. (**c**) Right bronchial lymph node metastasis. CD20 immunostained B lymphocytes in the inflammatory infiltrate of the tumor. ABC complex, ×400 magnification. (**d**) Right ovary. Desmoplasia and smooth muscle differentiation (arrow) are shown after Masson’s Trichrome staining, ×40 magnification. Inset: Details of multivacuolated or spongy neoplastic cells.

Immunohistochemical study showed high INHA expression in the tumor, suggesting SCST [6] (Figure 2c). This finding led us to exclude all type of carcinomas (including the one morphologically similar to SCST, i.e., clear cell carcinoma, which is INHA-negative) [18]. The germ cell origin of the neoplasia was also dismissed because the PLAP immunohistochemical stain showed very weak immunoreactivity, which was limited to scarce and isolated subsets of multinucleated giant neoplastic cells (Figure 2d). Ki-67 cell immunostaining determined an index proliferation lower than 5% (Figure 3a). In addition, immunohistochemistry confirmed inflammatory cell infiltration within the neoplastic masses, mainly composed of IBA1-positive macrophages and multinucleated giant cells (Figure 3b), and CD20-positive B lymphocytes (Figure 3c), indicative of chronic inflammation. T lymphocytes were rarely observed. The presence of desmoplasia and smooth muscle differentiation in the ovarian cell tumor (more evident in the right ovary) was confirmed by alpha-smooth muscle actin (SMA) immunohistochemistry and Masson’s Trichrome staining (Figure 3d).

## 4. Discussion

Based on histopathological and immunohistochemical studies, the ovarian tumor in the present study exhibited features of a bilateral SCST, specifically a SCT-NOS, as described by other authors [6,19]. To the best of our knowledge, it also represents the first report of an SCST in *Ursidae*.

SCT-NOS is a rare neoplasm with malignant potential, accounting for less than 0.1% of all human ovarian tumors [6,20], in contrast to granulosa cell tumor, which is considered the most common ovarian neoplasm in large animals [21]. Luteinized adult granulosa and thecoma cell tumors may show similar cytological features; therefore, they must be considered in the differential diagnosis [12,21]. In this regard, Call–Exner bodies and nuclear grooves, or “coffee bean”-like neoplastic cells, which are frequently described in granulosa cell tumors, were not observed in the studied bear tumor. A low level of PLAP expression can be found in different tumor entities [22], although it does not imply a germ cell neoplasia. In this line, the presence of few PLAP-positive multinucleated giant neoplastic cells, which is frequently observed in dysgerminomas [6], might suggest a limited component of neoplastic germ cells within the present SCT.

SCTs were previously known as “lipid” cell tumors until Scully [23] initiated the term “steroid cell tumors”, which best describes their morphological and functional structure. This nomenclature arises from their similarity to steroid hormone-secreting cells [24,25,26]. There are three subtypes of SCT: stromal luteoma (originated from the stroma), Leydig cell tumor (arising from the Leydig cells of the ovarian hilum), and SCT-NOS with controversial origin (likely derived from adrenal cortical rest cells, luteal stromal cells, or Leydig cells) [24]. The SCT-NOS differs from Leydig cell tumors because it lacks Reinke’s cytoplasmic crystals, and from stromal luteoma since the latter is associated with stromal hyperthecosis [6].

The malignant behavior of the bear tumor was confirmed considering the following gross (i) and microscopic (ii) pathological features: (i) significant large tumors with extensive abdominal spread and remote metastasis, which likely led to hemoperitoneum caused by abdominal blood vessels or spleen rupture, with subsequent hypovolemic shock; (ii) poor differentiation and pleomorphic neoplastic cells; high mitotic figures, including atypical mitosis; and areas of necrosis and hemorrhage [19,20]. Regarding the tumor microenvironment, the presence of macrophages and multinucleated giant cells was highly remarkable and should be emphasized, as they may have played an important role in tumor progression [27]. The tumor also displayed significant lipid accumulation (consequence of deregulation of lipid metabolism) and cholesterol crystals, both of which are related to malignant progression and increases in the growth and dissemination of tumor cells [28]. Testosterone is produced by most SCTs [6,26], but they can also cause hyperestrogenism [7,12,29] and hypercortisolism related to Cushing’s syndrome [6,30]. However, production of those hormones could not be confirmed in the studied bear. Moreover, the observed fibrosis and smooth muscle proliferation have previously been reported in other ovarian neoplasms, such as dysgerminoma and granulosa cell tumors [6,31,32].

SCT-NOS is usually found in adult females, although it also occurs in younger individuals and occasionally before puberty [19]. Specifically, the individuals tend to be younger than those showing Leydig cell tumors [12]; thus, based on research in human cancer, SCT-NOS can occur at all ages [6].

## 5. Conclusions

In conclusion, our findings are consistent with an ovarian SCT-NOS with malignant behavior, this being the underlying cause of death. Ultimately, however, hypovolemic shock led to the death of the animal, likely a consequence of tumor growth, which would have damaged the vessel walls.

## Figures and Tables

**Table 1:** Immunohistochemical procedures used for cell-type characterization.

Target Cell Type	Primary Antibody (Dilution ^a^)	Reference (Source)	Secondary Antibody (Dilution ^a^)	Positive Tissue Control
Stroma-cells ^b^	INHA, Inhibin-Alpha. Polyclonal (1:200)	PA5-95909 (Invitrogen-ThermoFisher Scientific. Waltham, MA, USA)	Goat anti-rabbit biotinylated (1:200)	Rabbit ovary
Germ-cells ^b^	PLAP, Placental Alkaline Phosphatase. Polyclonal (1:500)	PA5-78764 (Invitrogen-ThermoFisher Scientific. Waltham, MA, USA)	Goat anti-rabbit biotinylated (1:200)	Rabbit ovary
Macrophages ^b^	IBA1, Ionized Calcium-Binding Adapter Molecule. Polyclonal (1:1000)	019-19741 (FUJIFILM Wako Pure Chemical Corporation. Osaka, Japan)	Goat anti-rabbit biotinylated (1:200)	Badger lymph node
T Lymphocytes ^b^	CD3. Monoclonal (1:500)	NCL-L-CD3-565 (Leica Biosystems. Newcastle, UK)	Horse anti-mouse biotinylated (1:200)	Badger lymph node
B lymphocytes ^b^	CD20. Polyclonal (1:400)	PA5-16701 (Invitrogen-ThermoFisher Scientific. Waltham, MA, USA)	Goat anti-rabbit biotinylated (1:200)	Badger lymph node
Proliferative cells ^c^	Ki-67. Monoclonal (1:2000)	MIB-1. M7240 (DAKO. Glostrup, Denmark)	Horse anti-mouse biotinylated (1:200)	Sheep intestine
Smooth muscle cells and miofibroblasts ^c^	α-SMA, Actin Alpha-Smooth Muscle. Monoclonal (1:50)	1A4. A2547 (Sigma-Aldrich. Saint Louis, MO, USA)	Horse anti-mouse biotinylated (1:200)	Sheep intestine

^a^ Diluted in tris-buffered saline (TBS) with 0.1% bovine serum albumin (BSA). ^b^ Antigen retrieval with heat induction in sodium citrate buffer (pH 6) using a microwave (20 min at 700 watts). ^c^ Antigen retrieval with heat induction in sodium citrate buffer (pH 6) using a pressure cooker (3 min at full pressure).

## Data Availability

The data supporting the findings of this study are available from the corresponding author upon reasonable request.
